# MagIC-Cryo-EM: Structural determination on magnetic beads for scarce macromolecules in heterogeneous samples

**DOI:** 10.1063/4.0000912

**Published:** 2025-10-27

**Authors:** Yasuhiro Arimura

**Affiliations:** Fred Hutchinson Cancer Center, Seattle, WA, USA

## Abstract

High-resolution 3D structures of biomolecules, such as DNA and proteins, provide crucial insights into the chemical and mechanical mechanisms underlying their functions. However, the requirement for high sample concentrations (0.05–5.0 mg/ml) limits the applicability of structural analysis to only abundant targets. Additionally, determining the structure of small biomolecules (>200 kDa) remains challenging. To overcome these limitations, we developed two new cryo-EM-based methods: Magnetic Isolation and Concentration (MagIC)-cryo-EM and Duplicated Selection To Exclude Rubbish (DuSTER). The large sample requirement for cryo-EM arises from sample loss during the freezing step. We anticipated that this limitation could be overcome by locally enriching target biomolecules on nanomagnetic beads, which are small enough to be applied to cryo-EM specimens. To achieve this, we developed protein spacers designed for the direct structural analysis of targets captured on magnetic beads. Furthermore, we devised DuSTER, a method that excludes low signal-to-noise ratio particles, enabling the determination of small biomolecule structures. These methods enabled the cryo-EM structural determination of biomolecules in the 60–200 kDa range using low-concentration (0.0005 mg/ml) samples. These methods may significantly expand the applicability of cryo-EM, paving the way for researchers to elucidate the structural basis of biological events by visualizing biomolecules in their native environment.

